# Musculoskeletal conditions in children and adolescents managed in Australian primary care

**DOI:** 10.1186/1471-2474-15-164

**Published:** 2014-05-20

**Authors:** Nicholas Henschke, Christopher Harrison, Damien McKay, Carolyn Broderick, Jane Latimer, Helena Britt, Christopher G Maher

**Affiliations:** 1 The George Institute for Global Health, Sydney Medical School, University of Sydney, Sydney, Australia; 2 Institute of Public Health, University of Heidelberg, Heidelberg, Germany; 3 Family Medicine Research Centre, School of Public Health, University of Sydney, Sydney, Australia; 4 Children’s Hospital Institute of Sports Medicine, The Sydney Children’s Hospitals Network, Sydney, Australia; 5 School of Medical Sciences, The University of New South Wales, Sydney, Australia

**Keywords:** Musculoskeletal complaints, Primary care, Child, Adolescence

## Abstract

**Background:**

Primary care settings play a vital role in the early detection and appropriate management of musculoskeletal conditions in paediatric populations. However, little data exist regarding these conditions in a primary care context or on the presentation of specific musculoskeletal disorders in children. The aim of this study was to estimate the caseload and describe typical management of musculoskeletal conditions in children and adolescents presenting to primary care in Australia.

**Methods:**

An analysis of data from the Bettering the Evaluation and Care of Health (BEACH) study was performed. The BEACH study is a continuous national study of general practice (GP) activity in Australia. We identified all GP encounters with children and adolescents over the past five years and extracted data on demographic details, the problems managed, and GP management of each problem. SAS statistical software was used to calculate robust proportions and after adjustment for the cluster, the 95% confidence intervals (CIs).

**Results:**

From the period April 2006 to March 2011, there were 65,279 encounters with children and adolescents in the BEACH database. Of the 77,830 problems managed at these encounters, 4.9% (95%CI 4.7% to 5.1%) were musculoskeletal problems. The rate of musculoskeletal problems managed increased significantly with age, however there was a significant decrease for girls aged 15–17 years. Upper and lower limb conditions were the most common, followed by spine and trunk conditions. Spine and trunk conditions were significantly more likely to be managed with medication, but less likely to receive imaging, than upper or lower limb problems.

**Conclusions:**

Musculoskeletal problems in children and adolescents present a significant burden and an important challenge to the primary health care system in Australia. There is variability in rates of presentation between different age groups, gender and affected body region.

## Background

Musculoskeletal disorders are one of the most common causes of disability for people around the world [1]. In adults, musculoskeletal pain is a common reason for care seeking, especially in primary health care settings where it is typically assessed and managed [2]. Similarly, primary care settings play a vital role in the early detection and appropriate management of inflammatory and non-inflammatory musculoskeletal conditions in paediatric populations. Several studies have suggested a possible association between musculoskeletal pain and injury in childhood and development of musculoskeletal disorders in adults [3,4]. A better understanding of these conditions in children and adolescents is important for developing effective preventive strategies and to provide better understanding of the origin and progression of chronic pain into adulthood.

Numerous studies have shown that musculoskeletal pain is common in pre-adult populations [5,6]. Prevalence estimates vary by age and the case definition of musculoskeletal pain. In addition, most of the epidemiological studies on musculoskeletal pain and injury have been conducted either in a school setting [6], or with elite-level athletes [7]. In one of the only studies carried out in a primary care setting (in Spain), the prevalence of musculoskeletal pain was estimated to increase from 3% at the age of 3 years to 30% by age 14. This study reported that musculoskeletal pain is responsible for approximately 6% of the primary care visits of children between 3 and 14 years of age, and more than 10% of visits by adolescents [8]. In an earlier report of Australian general practitioner (GP) encounters with adolescents (aged 10–19 years), respiratory, skin, and musculoskeletal problems were found to be the most commonly managed problems [9]. A further investigation showed that musculoskeletal problems (including fracture, sprain and strain) were more likely to be managed in adolescent (12–18 years) boys than in girls [10].

In 2009–10 approximately 83% of the Australian population claimed at least one GP service from Medicare [11] with the average person visiting their GP 5.3 times between March 2010 and April 2011 [12]. In Australia, payment for GP visits is on a fee-for-service system with the majority of costs covered by Medicare, the universal Australian government funded medical insurance scheme. Still, little data exist in an Australian primary care context for younger children or on the presentation of specific musculoskeletal disorders. The objective of this study was to estimate the caseload and describe typical management of musculoskeletal conditions in children and adolescents managed in Australian general practice.

The specific aims were to: i) determine the rate at which musculoskeletal disorders were managed in children/adolescents by GPs in Australia; ii) identify the types of musculoskeletal disorders managed and differences between boys and girls, in different age groups; and iii) describe common management of musculoskeletal disorders by GPs in terms of medications prescribed, clinical treatments, diagnostic testing, and referrals made to specialists and allied health professionals.

## Methods

This is an analysis of data from the Bettering the Evaluation and Care of Health (BEACH) study. BEACH is a continuous national cross-sectional study of GP activity, involving ever-changing random samples of approximately 1,000 GPs per year (drawn by the Australian Government Department of Health and Ageing from insurance claims data). Each GP participant completes a questionnaire about themselves and their practice, and uses structured paper-based encounter forms to record details of 100 consecutive GP-patient encounters. This produces information for approximately 100,000 GP-patient encounters each year. Information collected includes: i) details about the encounter (e.g. date, payment method); ii) patient demographics (e.g. age, sex, postcode, ethnicity); iii) up to four diagnoses/problems managed; and iv) the management provided for each problem during the consultation (medications prescribed or supplied by the GP; clinical treatments such as general and specific advice, counselling or education, family planning, and administrative processes; procedural treatments including therapeutic actions and diagnostic procedures undertaken at the encounter; referrals to specialists and allied health services; and orders for pathology and imaging tests). Problems managed, clinical treatments, procedures, referrals and investigations are classified according to the International Classification of Primary Care-Version 2 (ICPC-2) [13] but coded more specifically with the Australian general practice clinical terminology, ICPC-2 Plus [14]. Completed encounter forms are returned to the BEACH research team for coding and data entry [12]. The BEACH database contains over 1.3 million records of GP-patient encounters collected since April 1998 from almost half of all the practising GPs in Australia [12].

Ethics approval for the BEACH program and its continued analysis was obtained for the Human Ethics Committee of the University of Sydney and the Ethics Committee of the Australian Institute of Health and Welfare. Oral informed consent was obtained from all parents or guardians of participants aged less than 18 years.

### Participants

We identified all GP encounters with children and adolescents aged less than 18 years recorded from April 2006 to March 2011 and identified those involving management of one or more musculoskeletal problems (as classified in Chapter L of ICPC-2). Each musculoskeletal problem managed was re-coded according to the broad location of the body area affected (upper limb, lower limb, spine and trunk, head and face), as “inflammatory” (for inflammatory musculoskeletal conditions), or as not otherwise specified. We then extracted demographic data on the patients and their GPs, and the management provided.

### Analysis

The BEACH study is a cluster sample design with a cluster of 100 patient encounters around each GP. We adjusted the 95% confidence intervals for the single stage clustered study design using procedures in SAS statistical software (version 9.1.3 SAS Inc, Cary, North Carolina). Due to the study design, statistical significance of differences is judged by non-overlapping 95% confidence intervals, which is more conservative than an alpha of 0.05. Results estimating the caseload of musculoskeletal problems are reported in terms of the rate per 100 GP encounters with children and adolescents. As more than one problem can be managed at each encounter, management actions (such as medication prescription) are reported as rates per 100 musculoskeletal problems managed.

## Results

In the period 2006 to 2011 there were 484,000 encounter records supplied by 4,840 GPs. Children and adolescents (aged <18 years) accounted for 65,279 (13.5%) encounters at which 77,830 problems were managed. Table 1 displays the ten most common problems managed (by ICPC-2 chapter) by GPs in children and adolescents during the study period. Musculoskeletal problems (n = 3,815) were the sixth most common type of problem managed in children and adolescents, accounting for 4.9% (95% CI 4.7%-5.1%) of all problems, and managed at a rate of 5.8 (95% CI: 5.6-6.1) per 100 encounters.

**Table 1:** Distribution of problems managed across ICPC-2 chapters in children and adolescents, 2006-11

**Problem**	**N**	**% of total problems**	**95% CI**
Respiratory	23686	30.43	(29.9-30.9)
General & unspecified	14932	19.19	(18.8-19.6)
Skin	11926	15.32	(15.0-15.6)
Ear	5902	7.58	(7.4-7.8)
Digestive	5551	7.13	(6.9-7.3)
*Musculoskeletal*	*3815*	*4.90*	*(4.7-5.1)*
Psychological	2495	3.21	(3.0-3.4)
Eye	2181	2.80	(2.7-2.9)
Endocrine & metabolic	1358	1.74	(1.6-1.9)
Neurological	1157	1.49	(1.4-1.6)
Other chapters	4827	6.2%	—
*Total*	*77830*	*100.0*	

**Figure 1:**
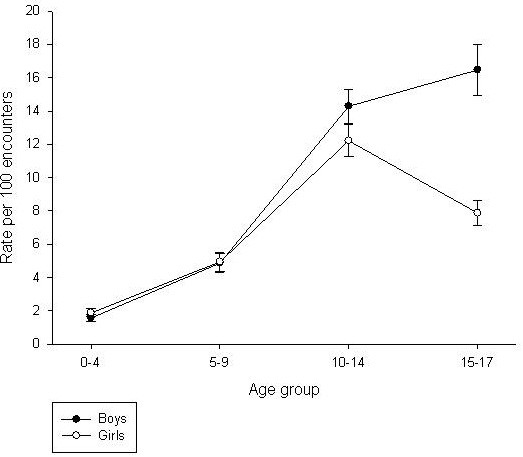
**Age-and sex-specific management rate of musculoskeletal problems managed by GPs.** Error bars indicate 95% confidence intervals.

There was a stepwise significant increase in the rate of musculoskeletal problems managed for both girls and boys from age 0–4 years to 5–9 years, and to age 10–14 years (Table 2). Across the two youngest age groups, there was no difference in management rates between the sexes. Boys aged 10–14 years and 15–17 years had a significantly higher management rate than girls of the same age. While there was no significant difference in the rate between boys aged 10–14 years and 15–17 years there was a significant decrease for girls aged 15–17 years (Figure 1). This group had a lower rate than both boys of the same age and girls aged 10–14 years.

**Table 2:** Age-sex specific management rate of musculoskeletal problems per 100 encounters

**Body region**		**Age group (years)**									
		**0-4 (n = 30,131)**		**5-9 (n = 13,383)**		**10-14 (n = 11,598)**		**15-17 (n = 9,638)**		**All† (n = 65,279)**	
		**Boys**	**Girls**	**Boys**	**Girls**	**Boys**	**Girls**	**Boys**	**Girls**	**Boys**	**Girls**
**Lower limb**	Number	99	110	118	121	311	254	169	134	697	619
	Rate/100 encs. (95% CI)	0.62 (0.50-0.74)	0.78 (0.63-0.93)	1.72 (1.39-2.06)	1.85 (1.51-2.19)	5.33 (4.72-5.95)	4.40 (3.85-4.96)	4.55 (3.84-5.26)	2.26 (1.87-2.65)		
**Upper limb**	Number	51	60	89	90	257	188	169	82	566	420
	Rate/100 encs. (95% CI)	0.32 (0.23-0.41)	0.42 (0.31-0.54)	1.30 (1.02-1.58)	1.38 (1.09-1.66)	4.41 (3.85-4.97)	3.26 (2.76-3.76)	4.55 (3.83-5.27)	1.38 (1.08-1.69)		
**Spine/trunk**	Number	25	11	43	39	108	104	108	122	284	276
	Rate/100 encs. (95% CI)	0.16 (0.09-0.22)	0.08 (0.03-0.12)	0.63 (0.43-0.83)	0.60 (0.40-0.79)	1.85 (1.49-2.21)	1.80 (1.43-2.17)	2.91 (2.32-3.49)	2.06 (1.68-2.44)		
**Head**	Number	22	12	2	4	13	10	21	16	58	42
	Rate/100 encs. (95% CI)	0.14 (0.08-0.19)	0.08 (0.04-0.13)	0.03 (0.00-0.07)	0.06 (0.00-0.12)	0.22 (0.10-0.34)	0.17 (0.07-0.28)	0.57 (0.31-0.82)	0.27 (0.14-0.40)		
**Inflammatory**	Number	2	10	3	7	4	7	4	11	13	35
	Rate/100 encs. (95% CI)	0.01 (0.00-0.03)	0.07 (0.03-0.11)	0.04 (0.00-0.09)	0.11 (0.03-0.19)	0.07 (0.00-0.14)	0.12 (0.03-0.21)	0.11 (0.00-0.21)	0.19 (0.07-0.30)		
**Not specified**	Number	54	63	78	63	140	142	141	102	413	370
	Rate/100 encs. (95% CI)	0.34 (0.25-0.43)	0.45 (0.33-0.56)	1.14 (0.88-1.40)	0.96 (0.69-1.24)	2.40 (1.95-2.86)	2.46 (2.00-2.92)	3.80 (3.06-4.54)	1.72 (1.35-2.09)		
**Total**	Number	253	266	333	324	833	705	612	467	2031	1762
	Rate/100 encs. (95% CI)	1.58 (1.38-1.78)	1.88 (1.65-2.12)	4.87 (4.31-5.42)	4.95 (4.39-5.52)	14.29 (13.26-15.31)	12.22 (11.25-13.20)	16.47 (14.95-18.00)	7.88 (7.14-8.63)		

†Patient sex was not recorded for six 5–9 year olds, eight 10–14 year olds and eight 15–18 year olds.

When the management rates of problems related to specific body locations are examined, a stepwise significant increase is seen in upper and lower limb musculoskeletal conditions up to the 10–14 year age group (Table 2). Girls aged 15–17 years had a significantly lower management rate of upper (1.38 per 100 encounters) and lower limb conditions (2.26 per 100) than boys of the same age (4.55 per 100 for both) and girls aged 10–14 years (3.26 and 4.40 per 100). The management rate of spine and trunk problems in boys showed a significant increase with each age group. However, there was no significant difference between the sexes in any age group for spine and trunk problems. The management rate of inflammatory musculoskeletal conditions was extremely low across all age groups and both sexes.

The GP management of musculoskeletal conditions in children and adolescents, split by sex and age group, is described in Table 3. Children in the youngest age group (0–4 years) were significantly less likely to have their musculoskeletal problem managed with medication. Girls aged 15–17 years were more likely to receive medication than those aged 5–9 years (39.40 vs 25.31 per 100 musculoskeletal problems managed). Procedural treatments for musculoskeletal conditions were also less common in the youngest age group than in older children. Specialist referral was more likely among girls aged 0–4 years (15.41 per 100 musculoskeletal problems managed) than those aged 5–9 years (5.56 per 100) or 10–14 years (6.52 per 100). The rate of referral to allied health services for both girls and boys in the oldest age group (15–17 years) was significantly higher than it was in the youngest two age groups.

**Table 3:** GP management of musculoskeletal problems in children: rate per 100 musculoskeletal problems managed (95% CI)

**Management**	**Age group (years)**							
	**0-4**		**5-9**		**10-14**		**15-17**	
	**Boys (n = 253)**	**Girls (n = 266)**	**Boys (n = 333)**	**Girls (n = 324)**	**Boys (n = 833)**	**Girls (n = 705)**	**Boys (n = 612)**	**Girls (n = 467)**
**Medication**	12.65 (8.40-16.90)	14.66 (9.84-19.48)	24.92 (19.78-30.07)	25.31 (19.82-30.80)	25.57 (22.09-29.05)	32.62 (28.40-36.85)	32.52 (27.93-37.10)	39.40 (34.03-44.77)
***Prescribed***	5.53 (2.50-8.56)	5.26 (2.38-8.14)	6.01 (3.19- 8.82)	7.41 (4.18- 10.64)	9.24 (6.87-11.61)	9.50 (7.01-12.00)	15.69 (12.49-18.89)	17.77 (13.91-21.63)
***Advised OTC*** ***†***	6.72 (3.61-9.83)	7.89 (4.19-11.60)	18.02 (13.55-22.49)	16.36 (11.65-21.06)	14.89 (12.19-17.58)	21.56 (18.04-25.08)	14.54 (11.30-17.79)	18.63 (14.67-22.59)
***GP supplied***	0.40 (0.00-1.17)	1.50 (0.03-2.98)	0.90 (0.00-1.92)	1.54 (0.19-2.90)	1.44 (0.57-2.31)	1.56 (0.42-2.70)	2.29 (0.94-3.64)	3.00 (0.86-5.13)
**Other treatments**	41.11 (34.11-48.10)	33.08 (26.57-39.60)	46.25 (39.29-53.20)	45.37 (38.09-52.65)	47.30 (42.58-52.02)	50.21 (43.92-56.50)	45.10 (39.23-50.97)	47.54 (41.15-53.93)
***Clinical‡***	27.67 (21.74-33.60)	20.30 (15.03-25.57)	21.02 (16.23-25.81)	23.46 (18.76-28.16)	24.01 (20.70-27.32)	24.26 (20.20-28.41)	23.04 (18.62-27.46)	25.48 (20.85-30.11)
***Procedural§***	13.44 (8.68-18.20)	12.78 (8.42-17.15)	25.23 (19.77-30.68)	21.91 (16.32-27.50)	23.29 (19.96-26.62)	25.96 (21.61-30.31)	22.06 (18.21-25.91)	22.06 (17.44-26.68)
**Referrals**	19.37 (14.08-24.65)	23.68 (18.20-29.17)	16.22 (12.03-20.40)	12.35 (8.65-16.05)	16.93 (14.25-19.60)	16.45 (13.71-19.20)	23.53 (19.79-27.27)	23.13 (19.09-27.16)
***Hospital***	1.98 (0.25-3.70)	1.13 (0.00-2.41)	0.60 (0.00-1.43)	1.13 (0.00-2.41)	1.08 (0.37-1.79)	0.57 (0.02-1.12)	0.82 (0.10-1.53)	0.21 (0.00-0.63)
***Specialist***	12.25 (7.95-16.55)	15.41 (11.00-19.83)	8.41 (5.27-11.55)	5.56 (2.90-8.21)	7.32 (5.48-9.17)	6.52 (4.66-8.39)	10.95 (8.41-13.48)	10.06 (7.26-12.87)
***Allied health services***	4.35 (1.82-6.87)	5.26 (2.36-8.16)	5.71 (2.98-8.43)	4.94 (2.57-7.31)	7.44 (5.59-9.30)	8.37 (6.32-10.41)	11.11 (8.53-13.70)	11.78 (8.76-14.80)
***Emergency department***	0.79 (0.00-1.89)	0.75 (0.00-1.79)	1.50 (0.19-2.81)	1.85 (0.38-3.33)	0.84 (0.22-1.46)	0.57 (0.01-1.12)	0.16 (0.00-0.48)	0.43 (0.00-1.02)
**Imaging**	35.57 (28.85-42.30)	36.47 (30.01-42.92)	35.74 (29.57-41.90)	41.05 (34.70-47.40)	43.46 (39.45-47.46)	37.30 (33.11-41.50)	41.67 (37.18-46.16)	34.05 (29.13-38.97)
**Pathology**	5.14 (0.00-10.89)	6.02 (0.94 -11.09)	13.21 (3.12-23.31)	7.72 (2.18-13.26)	4.32 (1.54-7.11)	9.93 (4.58-15.28)	12.91 (6.65-19.17)	12.85 (6.30-19.40)

†OTC – over the counter.

**‡**Clinical treatments include general and specific advice, counselling or education, family planning, and administrative processes.

§Procedural treatments include therapeutic actions and diagnostic procedures undertaken at the encounter.

The GP management in the three main body location groups (lower limb, upper limb, spine and trunk) is described in Table 4. Children and adolescents with spine and trunk conditions were significantly more likely to be managed with medication, but less likely to receive imaging, than those with upper or lower limb problems. Upper limb conditions had a significantly greater rate of procedural treatments, emergency department referrals, and imaging tests than the other two groups. A lower rate of referral to allied health services and ordering of pathology tests was also seen in upper limb conditions.

**Table 4:** GP management of musculoskeletal problems by body location in children rate per 100 musculoskeletal problems managed (95% CI)

**Management**	**Body location**		
	**Upper limb (n = 990)**	**Lower limb (n = 1323)**	**Spine or trunk (n = 564)**
**Medication**	19.49 (16.60-22.39)	22.98 (20.37-25.58)	45.05 (40.06-50.01)
***Prescribed***	5.96 (4.35-7.57)	9.07 (7.33-10.81)	17.91 (14.22-21.59)
***Advised OTC†***	12.22 (9.81-14.64)	12.55 (10.57-14.53)	24.65 (20.76-28.53)
***GP supplied***	1.31 (0.61-2.02)	1.36 (0.55-2.17)	2.48 (0.89-4.08)
**Other treatments**	48.79 (44.68-52.90)	44.07 (40.30-47.83)	44.15 (38.58-49.71)
***Clinical‡***	14.04 (11.62-16.46)	27.59 (24.75-30.43)	24.11 (20.17-28.06)
***Procedural§***	34.75 (31.33-38.17)	16.48 (14.06-18.90)	20.04 (15.37-24.70)
**Referrals**	13.84 (11.45-16.22)	21.47 (19.16-23.77)	19.68 (16.32-23.04)
***Hospital***	1.41 (0.68-2.15)	0.68 (0.24-1.12)	0.53 (0.00-1.13)
***Specialist***	6.06 (4.49-7.63)	9.52 (7.88-11.17)	6.91 (4.82-9.01)
***Allied health services***	4.24 (2.89-5.59)	10.36 (8.67-12.04)	11.70 (8.99-14.41)
***Emergency department***	1.72 (0.86-2.57)	0.45 (0.09-0.82)	0.35 (0.00-0.85)
**Imaging**	56.67 (53.03-60.30)	40.21 (36.98-43.45)	25.00 (21.03-28.97)
**Pathology**	0.71 (0.00-1.77)	7.18 (4.03-10.33)	7.27 (2.30-12.24)

†OTC – over the counter.

**‡**Clinical treatments include general and specific advice, counselling or education, family planning, and administrative processes.

§ Procedural treatments include therapeutic actions and diagnostic procedures undertaken at the encounter.

## Discussion

This study has provided the first description of the primary care management of musculoskeletal conditions in Australian children and adolescents. In children less than 18 years old musculoskeletal problems are commonly managed in primary care, at a rate of 5.8 (95% CI: 5.6-6.1) per 100 encounters. This can be extrapolated to an estimate of 880,000 musculoskeletal problems in children and adolescents managed per year in Australia [11]. For both boys and girls, the rate of musculoskeletal problems managed increased significantly with age. There were no differences between boys and girls of the same age, except for the 15–17 year old age group, where a significant decrease in the management rate of musculoskeletal conditions was seen in girls. Upper and lower limb conditions were the most common, followed by spine and trunk conditions. Medication was more often prescribed to girls in the oldest age group and referral to allied health was also more common in the older age groups. When compared to upper and lower limb problems, children and adolescents with spine and trunk conditions were more likely to be managed with medication but less likely to receive imaging.

One explanation for the observed increase in management rate of musculoskeletal conditions with age is that this coincides with a critical period of growth and development for the musculoskeletal system. It has been hypothesised that this can be attributed to the increased physical fragility associated with the pubertal growth spurt or a heightened attentiveness to somatic symptoms as a result of rapid physical growth [15]. During puberty, bone mineral is deposited at an accelerated rate under the influence of gonadal steroids, with peak bone mass achieved by early adulthood [16]. Major musculoskeletal problems such as adolescent idiopathic scoliosis and slipped capital femoral epiphysis are linked to peak growth velocity during puberty [15]. Other conditions which have a peak onset around puberty are the osteochondroses (conditions which affect growing bones and articular cartilage) such as Scheuermann’s disease, Freiberg’s disease, and osteochondritis dissecans. These conditions can result in significant discomfort and reduction in physical activity and sport participation, as well as being associated with a higher risk of long term disability and deformity [17].

The variability of problems across body locations and age groups has highlighted a need for further understanding of the aetiology of musculoskeletal complaints in children and adolescents. One limitation of the current study is that little data were available on whether the problems managed were in an acute or chronic phase, this information being important for understanding the possible aetiology of these complaints. Previous studies have reported a prevalence of chronic musculoskeletal pain in school children of up to 40% [18]. It has also been suggested that up to 40% of children with musculoskeletal complaints still have problems four years later [19] and that musculoskeletal conditions in childhood substantially increase the risk of further musculoskeletal conditions in adulthood [3,20]. As chronic musculoskeletal pain is becoming an increasing public health concern, effective prevention and appropriate management strategies could potentially be of most benefit when started early, being focused on children and adolescents within the primary care setting.

One proposed avenue for reducing the burden of musculoskeletal complaints in children and adolescents is through sports injury prevention [7]. Physical trauma, including sports injuries, has been shown to be the most common aetiology for pain among adolescents visiting a primary care physician [8]. Children who frequently exercise or participate in organised sport have an increased risk of musculoskeletal injuries and more often seek care for musculoskeletal pain [21]. It is estimated that 8% of adolescents in Australia drop out of recreational sporting activities annually because of injury [22], which can have large public health implications as both physical activity [23] and obesity [24] have been shown to track from childhood to adulthood. The observed reduction in management rates for 15–19 year old girls in the current study, especially for extremity complaints, may be related to the fact that adolescent girls are more likely to drop out of recreational sporting activities than boys [22]. In order to balance the risks with the benefits of regular physical activity and sports participation, further research is needed to evaluate the causes of musculoskeletal complaints as well as the implementation of prevention strategies in youth sport [7].

In Australia, general practice is the cornerstone of primary health care and young people nominate GPs as their health provider of choice. However, a previous study which used the BEACH database identified that adolescents (12–18 years) were under-represented in general practice consultations. It was found that between 1999 and 2004 adolescents in Australia made up only 4.0% of general practice encounters, but 9.7% of the population [9]. The same study also showed that the encounter rate was substantially higher among 15–19 year old girls compared with 15–19 year old boys. The authors noted that 80% of this difference was accounted for by girls of this age group having higher urological and reproductive health issues as their reasons for encounter [9]. This could explain part of the significant drop in the management of musculoskeletal problems among girls aged 15–17 years, with their increased management of reproductive issues effectively lowering their management rate of musculoskeletal problems on a per encounter basis, relative to the boys and the younger age groups.

The substantial burden of musculoskeletal complaints in young people presenting to GPs dictates a need for a distinct evidence base to inform primary care management in children and adolescents. In the current analysis, significant differences were observed in medication prescription, imaging, and referral to allied health services across age groups and by location of the complaint. This variability is difficult to explain and may result from the lack of evidence-based recommendations for management of musculoskeletal conditions in children and adolescents. While the rate of inflammatory conditions (such as juvenile idiopathic arthritis) managed was very low across all age groups, most inflammatory musculoskeletal conditions require specialist management and early diagnosis is vital [25]. Delayed diagnosis and access to specialised therapy of inflammatory conditions in children and adolescents has been recognised as detrimental to long term outcomes [26]. The GP is often the first care provider consulted with symptoms related to these conditions and it is in primary care that accurate screening tools are needed to alert clinicians to the need for further referral.

## Conclusions

Musculoskeletal problems in children and adolescents present a significant caseload and an important challenge to the primary health care system in Australia. The variability in rates of presentation between age groups and affected body region highlight a need for a specific evidence-base to support GP management of these conditions.

## Abbreviations

BEACH: Bettering the Evaluation and Care of Health; GP: General practice; ICPC-2: International Classification of Primary Care, Second Edition; OTC: Over the counter.

## Competing interests

All authors declare that they have no competing interests.

## Authors’ contributions

NH was involved in the conception and design of the study, interpretation of data, wrote the first draft and revised the paper. He is the guarantor. CH and HB were involved in the conception and design of the study, performed the data analysis, and critically revised the paper. DM, CB, JL, and CM were involved in the conception and design of the study, interpretation of data, and critically revised the paper. All authors gave approval to the final version of the submitted paper.

## Pre-publication history

The pre-publication history for this paper can be accessed here:

http://www.biomedcentral.com/1471-2474/15/164/prepub
